# A Comparative Analysis of the Fatigue Strength of Aluminium and Copper Wires Used for Power Cables

**DOI:** 10.3390/ma18184426

**Published:** 2025-09-22

**Authors:** Tadeusz Knych, Beata Smyrak, Bartosz Jurkiewicz

**Affiliations:** Faculty of Nonferous Metals, AGH University of Krakow, 30-059 Krakow, Poland

**Keywords:** fatigue, aluminium fatigue, copper fatigue, wires, power lines, offshore cables

## Abstract

Recent studies have demonstrated that the utilisation of aluminium in electrical applications has increased substantially, particularly in the context of power cables. The substitution of copper with aluminium in cable fabrication is predominantly driven by economic considerations. When designing such cables, it is imperative to ascertain their functional properties, including their electrical conductivity and mechanical properties, and their operational properties, which include rheological, thermal, and material fatigue resistance. This is to ensure that the aluminium and copper cables are compatible. The primary challenge confronting researchers in this domain pertains to predicting and forecasting the failure of overhead cables during their operational lifecycle. One of the most significant and prevalent operational hazards is fatigue damage. This article presents the experimental results of fatigue tests on single Al and Cu wires in various states of mechanical reinforcement. The parameters of the Wöhler curve were determined, and a comparative analysis of the morphology of fatigue damage in single copper and aluminium wires was performed. It was found that copper wires are more fatigue-resistant than aluminium wires. In the case of high-cycle fatigue, this difference can amount to 10^6^ cycles. An analysis of fatigue fracture morphology showed that fractures have a developed surface and that plastic deformation makes a significant contribution in the case of low-cycle fatigue. In the case of high-cycle fatigue, many cracks were observed in the copper wires. No such cracks were observed in the aluminium wires.

## 1. Introduction

The cable industry is currently dominated by trends relating to two main factors: First, there is a need to improve the performance of cables and wires, which is driven by a demand for their use in new applications. Second, the global economic situation determines the optimal production process. The development of new applications, including the production of electricity from renewable energy sources, modern aviation and automotive technologies, and electronics and telecommunications, necessitates the implementation of cables with novel designs and structures, accompanied by technological advancements that satisfy contemporary requirements, particularly in electrical power transmission and distribution systems. The need to minimise spatial requirements and mass, in conjunction with the escalating demand for electricity, necessitates the optimisation of cable geometry (i.e., shape and size). Concurrently, this necessitates the assurance of enhanced physical properties and economical operation. It is only possible to meet these requirements through the continuous development of new materials and the modification of cable and wire production technologies. Conversely, the ultimate selection of novel technological solutions is contingent upon prevailing global economic conditions, particularly within the raw materials market [1]. The elevated and fluctuating cost of copper, which was evident in the preceding decade, has precipitated transformative shifts in the configuration of contemporary cables and wires. This has entailed the adoption of aluminium and aluminium-based alloys as fundamental raw materials. This situation has led to rapid developments in the technology used to manufacture aluminium alloys for electrical applications and their processing into cables and wires [2,3].

The substitution of copper with aluminium in electrical applications has been a discernible trend for approximately a decade, exhibiting a consistent upward trajectory and an irreversible nature in numerous products. At present, approximately 15% of global aluminium production is utilised for electrical applications. The largest share is accounted for by large products, i.e., cables and power cables. Consequently, the development of aluminium and its alloys in power transmission and distribution will account for a significant share of aluminium’s electrical applications.

In the context of using aluminium in electrical applications, it is imperative to consider not only its functional properties (i.e., electrical conductivity and mechanical properties) but also its operational properties, such as its creep resistance, corrosion resistance, heat resistance, and fatigue resistance. In the field of electrical engineering, the design of overhead lines is of paramount importance. A key consideration in this design process is the resistance of the lines to fatigue. This is also a significant factor in the design of cable systems, including submarine cables.

**Figure 1:**
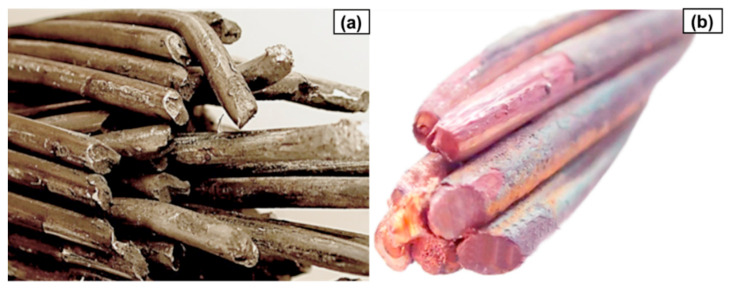
Photos of fatigue wear of aluminium conductor steel-reinforced (ACSR) conductors (**a**) and copper conductors from a railway network (**b**) used in overhead lines (own research).

Figure 1 presents photos of examples of aluminium and copper conductors’ fracture.

In both scenarios, the focus is on dynamic cable operation, which is characterised by the movement of wind in the case of overhead conductors and the constant movement of seawater and waves in the case of offshore cables. In the context of these applications, simulation tests are of paramount importance. Research has indicated that wind is responsible for approximately 25% of all documented failures. This phenomenon is the result of the dynamic interaction between wind and power lines, causing vibrations of varying amplitudes and frequencies.

Wind-induced vibrations, which are characterised by a low amplitude and high frequency, are the most dangerous and occur mainly in flat, low-lying areas. The vibrations in question typically possess an amplitude that is smaller than the diameter of the cable, with a frequency ranging from a few Hertz to over 100 Hz. It has been established that repeated cycles of such vibrations occurring during line operation are the primary cause of wire breakage near the point where the cable exits the fasteners. This phenomenon can be considered a superposition of the classic process of material fatigue and fretting (also known as friction corrosion, friction slip, or friction oxidation). This process can be defined as the grinding of wire surfaces moving relative to each other. It has been demonstrated that, over time, these vibrations can lead to material fatigue processes spreading throughout the cable components. This, in turn, can result in breaks and, consequently, overhead line failures. This phenomenon can be attributed to the combined effect of various phenomena that cause localised damage and reduce the active surface area of the cable cross-section. This results in a disruption to the flow of the current, leading to a local increase in temperature. Furthermore, fatigue cracks have been demonstrated to reduce the cable’s mechanical strength, and in extreme cases, they can result in a cable’s complete destruction [4,5,6].

It has been demonstrated that the effects of wind vibrations can be mitigated by passive vibration protection, which involves reducing the voltage, and active vibration protection, which involves the use of dampers. It is evident that wind vibration exerts a substantial influence on cable tension, thereby imposing considerable constraints on the system [7,8,9,10]. Material fatigue in overhead lines is frequently observed in proximity to suspension clamps or other devices attached to the cable.

The suspension clamp is of particular significance in the context of wire fatigue, given the numerous loads to which the wire/clamp assembly is subjected. These include tensile load on the conductor, bending displacement resulting from wind vibrations, and clamping torque [11,12,13].

In recent decades, the most significant challenge relating to copper cables has been their application in wind farms and floating offshore wind turbines (FOWTs) [14]. In such cases, it is imperative that cables are subjected to rigorous testing to ascertain their suitability for deployment. It is also imperative that this testing encompasses a range of environmental factors, including exposure to sunlight, wind, wave loads, and sea water pressure. In particular, floating offshore wind turbines are subjected to wind, current, and wave loads, which result in variable and continuous dynamic movements and stresses in the power cables connecting individual FOWTs to each other and to land. The primary cause of fatigue damage is the accumulation of variable stresses in cables [14,15,16].

The findings of this study indicated that material fatigue was the most prevalent cause of failure in offshore cables, a phenomenon analogous to that observed in overhead power lines. Marta et al. [17] presented the results of tests conducted on power cables that were installed in several floating offshore locations. The findings of this study indicated that the presence of interlayer friction (fretting) has a significant impact on the deterioration of copper conductors and contributes to the exacerbation of fatigue damage. Various studies have predicted power cable fatigue in simulations. For instance, in [18,19], a cyclic stress curve (S-N) for a copper dynamic marine power cable was developed, and the influence of bending and tensile loads on the performance of copper conductors was analysed using a numerical calculation. A substantial amount of research has been performed on dynamic motions and mechanical properties [20,21]. The findings of this research indicate that cable curvatures generally attain their maximum extent at high waves and during the wave resonance period. In a related study, Thies examined the impact of diverse wave configurations, such as “lazy waves” and “chain waves”, on the fatigue strength of cables. The authors of [22] conducted a study on the behaviour of a submarine power cable with a copper conductor when subjected to dynamic bending. It was determined that the primary factor contributing to fatigue damage is the nonlinear bending of the power cable and that, at this stage, the primary factor contributing to fatigue damage is the local friction effects between the strands in the cable. In [17,23], models were developed to predict the fatigue life of dynamic power cables through the utilisation of simulations and experiments.

The primary challenge lies in the prediction and forecasting of overhead cable failures during their service life, which, in accordance with current standards, should span a minimum of 50 years without failure.

In order to predict the service life of cables, it is necessary to analyse both the results of experiments and actual cases of cable damage. The latter are frequently the consequence of a combination of material fatigue, friction, and other types of wear, including abrasion and thermal wear. In addition, experimental fatigue strength tests are conducted on a range of materials, which are then utilised when cables are designed and manufactured. The objective of this study is to ascertain the relationship between the cyclic load on the sample and the number of cycles until the onset of cracking. The most widely employed analysis method is the Wöhler curve, which illustrates the relationship between stress and the number of cycles required to destroy a sample [24]. The construction of such a curve involves determining the fatigue of test samples at a specific stress amplitude (σ_a_), which remains constant throughout the fatigue test until failure occurs after a specified number of cycles (N). This process is then repeated at decreasing stresses to cause the sample to fracture over a longer period of time and obtain an S-N curve (stress amplitude as a function of the number of cycles) [25,26,27].

The fatigue strength of cables is dependent on the type of material, specific cable construction (number and diameter of wires and length of lay), and operating parameters of the particular cable, such as stress, temperature, wind speed, and environment.

Cables consist of wires that are in various states of hardening, including soft (annealed), semi-hard, and hard. The strength range of copper wires spans from 200 to 430 (MPa), while aluminium wires have a strength range spanning from 60 to 180 MPa. Furthermore, it is imperative that the wires exhibit minimum electrical conductivity (58 MS/m for Cu wires and 35 MS/m for Al wires) [28,29,30,31,32,33].

Overhead conductors or cables, composed of copper or aluminium, are constructed from wires that exhibit varying degrees of mechanical hardening. In view of the prevalent trend of substituting copper cables with aluminium ones, it is imperative to ascertain the experimental disparity in fatigue strength between aluminium and copper wires and determine the influence of strain hardening (SH) on fatigue life.

## 2. Materials and Methods

### 2.1. Materials

Two types of wire were tested: EN AW-1370-grade aluminium wires used for overhead power conductors and Cu-ETP-grade copper wires used in cables. The chemical composition of the materials is presented in Table 1 (aluminium) and Table 2 (copper).

**Figure 2:**
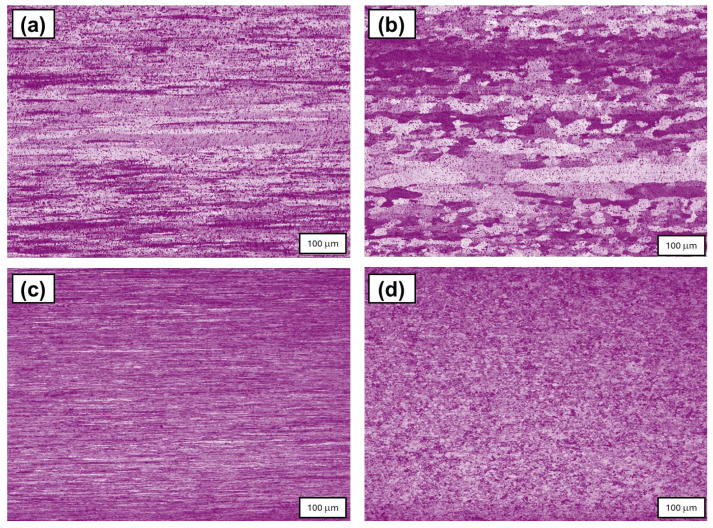
Comparison of optical microscopy images of the longitudinal cross-section wires with a diameter of 3.00 mm, (**a**) EN AW-1370 wires (90% SH), (**b**) EN AW-1370 wires (5% SH), (**c**) Cu-ETP wires (90% SH), and (**d**) Cu-ETP wires (5% SH).

Figure 2 presents the microstructure of the cross-section of the wires. The predominant distinction in microstructure is linked to the dimensions of grains and the existence of impurities. Cu-ETP has a grain size of approximately 20 micrometres, while EN AW-1370 has a grain size of 100 micrometres. Furthermore, the Al samples demonstrate more precipitations, resulting from the 0.1% content of Fe by weight and 0.03% wt. content, which occurs in the form of Al-Fe-Si.

The wires were found to be in two different mechanical states, i.e., in various states of strain hardening (SH): (a) engineering strain = 5% and (b) engineering strain = 90%. The procedure involved the process of wiredrawing wires with different initial diameters to wires with a final diameter of 3 mm. Wires with different initial diameters were pre-annealed to achieve the same level of mechanical properties. The level of strain hardening (SH) of the wires after the wiredrawing process is presented as the engineering strain (*ε_A_*) according to the following formula:

(1) $$\varepsilon_{A}=\frac{A_{0}-\;A_{1}}{A_{0}}$$

where

$A_{0}$—cross-section of the wire before the wiredrawing process;
$A_{1}$—cross-section of the wire after the wiredrawing process.

The mechanical properties of these wires are presented in Table 3.

### 2.2. Equipment and Research Methodology

**Figure 3:**
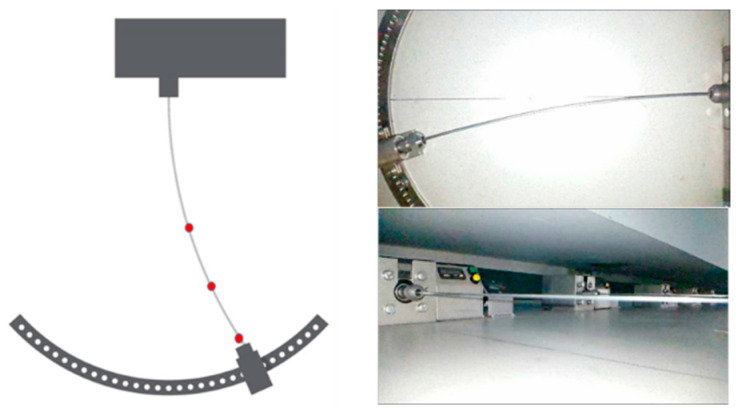
Photo of the wires in the fatigue testing stand.

Fatigue strength tests were conducted on a rotating bending testing apparatus, which induces variable stress in the material by bending the sample. The wire undergoes an axial rotational motion at a speed of 3000 RPM. Stress modification is achieved by symmetrically bending the sample to a known deflection (see Figure 3).

The procedure for preparing samples for fatigue testing involved creating straight samples of approximately 400 mm in length, which were then mounted on a rotating bending testing apparatus to assess the material’s fatigue strength.

Measurements of the number of cycles to destruction were taken on three occasions for each level of stress, and subsequently, the mean number of cycles was calculated. The predominant method used to analyse the results obtained from the fatigue tests involves preparing what are known as fatigue characteristics, which are commonly referred to as “S-N” diagrams or Wöhler diagrams.

These diagrams are obtained by plotting measurement points (the average number of cycles from multiple measurements until sample failure at a given stress) on a σ-N graph (stress–number of cycles) and connecting them with a curved line. As demonstrated in the existing literature, Wöhler graphs have historically been presented in three different coordinate systems: σ−N, σ−logN, and logσ−logN.

“S-N” charts enable a direct comparison of the fatigue strength of selected materials. Furthermore, they facilitate the comparison of the tested materials with data from the literature. The mean value of the number of cycles for a given stress constituted the basis for the development of the Wöhler curve. The determination of a single Wöhler curve necessitated approximately 30 measurements. Subsequently, Wöhler curves were utilised to further analyse the results regarding the material’s fatigue resistance.

The wires were subjected to fatigue tests, with stress values being recorded according to the data presented in Table 4.

The bending stress was determined based on the following relationship:

(2) $$\sigma_{g}=\frac{3\cdot E\cdot d\cdot y.}{2\cdot L^{2}}$$

where

$\sigma_{g}$—max bending stress [MPa];
E—Young’s modulus [GPa];
d—wire diameter [mm];
y—wire deflection arrow [mm];
L—wire length [mm].

The morphology of wire fractures post-fatigue was examined via scanning microscopy (SEM) using a Hitachi SU-70 high-resolution scanning electron microscope (Hitachi, Tokyo, Japan). The accelerating voltages (AVs) were set at 15 kV.

The SEM test was conducted at a range of magnifications across various fracture areas, with the objective of identifying fatigue striations and regions of plastic deformation and fracture, as well as fatigue and fracture separation.

## 3. Results and Discussion

Based on the methodology outlined above, we performed research on the fatigue process of aluminium and copper wires in various states of hardening. The obtained results regarding the number of cycles to failure for a given stress are presented in Table 3. We also performed basic tests of the fatigue process of aluminium and copper wires in various states of hardening, and the results obtained, in the form of the number of cycles to failure for a given stress, are presented in Table 5.

The subsequent analysis of the results enabled the development of the so-called Wöhler curves, a mathematical description of the relationship between stress and the number of cycles to failure, followed by an analysis of the wires for fractures after the fatigue process.

### 3.1. Phenomenological Analysis of Wire Fatigue Results

**Figure 4:**
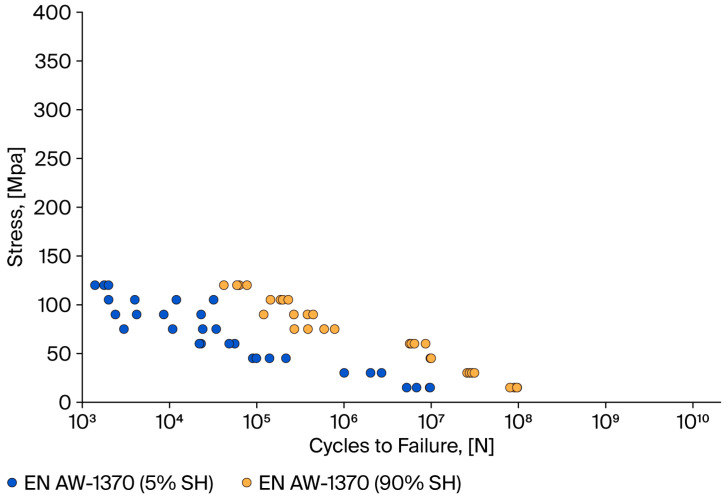
Relationship between medium stress and cycles to failure of aluminium wires in different mechanical states.

**Figure 5:**
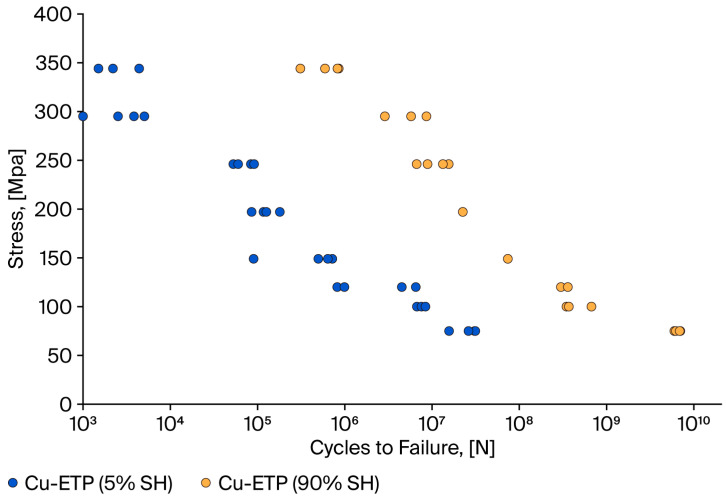
Relationship between stress and number of cycles to failure of copper wires in different mechanical states.

Based on the results obtained (see Table 5), Wöhler characteristics, i.e., the relationship between the average stress and the number of cycles to wire failure in the fatigue test, were developed. The results are presented in Figure 4 (aluminium wires) and Figure 5 (copper wires).

As demonstrated in Figure 4, the fatigue strength of Al wires with 90% SH and for a stress of 15 MPa is 10^8^, while for annealed wires, it is 10^7^ cycles. The present study’s findings are consistent with those reported in previous research conducted by the authors [24,34,35], which focused on wires [28] and wires and cables [34,35].

A comprehensive evaluation of the fatigue strength indicates that, for cold-drawn wires of copper (90% SH), the number of cycles to failure is approximately 10^10^, as indicated by a sigma max value of 75. For wires with 5% SH, the number is 10^7^. These results are consistent with those reported in [34], wherein the authors conducted experimental tests on copper wires in a hardened and annealed state (600 °C/1 h). Specifically, for hardened and annealed wires, the fatigue strength is equivalent to 10^9^ under a stress of 75 MPa. In turn, in [33], the authors studied oxygen-free copper in two mechanical states: annealed (850 °C)/20 min and strengthened. For a stress of 50 MPa, the number of cycles was 10^7^. Similar results were obtained by the authors of [36,37,38], where for a stress of 75–80 MPa, the number of cycles to fatigue was 10^7^.

In order to perform a comparative analysis of the fatigue strength of copper and aluminium, it is necessary to take the differences in their strength properties into account. Therefore, the strength ratio was determined. The strength factor is defined as the ratio of the maximum stress that is measured during a fatigue test to the yield strength of the wires under consideration.

The strength factor employed for the purposes of our analysis in this study is defined as follows:

(3) $$W_{f}=\frac{\sigma_{max}}{Y_{p}}$$

where

$W_{f}$—strength factor [%];
$\sigma_{max}$—max stress of rotating bending fatigue test [MPa];
$Y_{p}$—yield point of wires [MPa].

**Figure 6:**
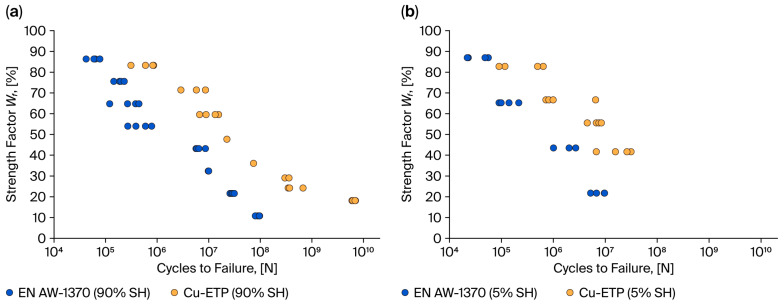
Comparison of strength factor, $W_{f}$, and the cycles to failure of aluminium and copper wires, (**a**) wires in a 90% SH, and (**b**) wires in a 5% SH.

Figure 6 shows the relationship between the strength factor and cycles to failure for aluminium and copper wires.

A thorough examination of the findings reveals that, in the context of copper (Cu) wires, the fatigue test results fall into two distinct groups: one for hardened wires and one for soft wires. Conversely, the fatigue test results for aluminium (Al) wires in both the hardened and soft states manifest as a set of overlapping points (see Figure 5). Furthermore, a substantial increase in the variability of the individual fatigue test results for aluminium wires is evident. For a given level of stress, the number of cycles to failure can vary by up to one million. As demonstrated in Figure 6, the results of the fatigue test permit a direct evaluation of the fatigue properties of copper and aluminium wires. For example, for a strength factor value of 25%, the fatigue strength for Al-hardened wires is 10^7^–10^8^ cycles, while for Cu-ETP wires, it is 10^9^ cycles. The wires with a hardening level of 5% and a strength coefficient value of 45% achieve a cycle range of 10^6^ to 10^7^ for aluminium wires and 10^8^ to 10^9^ for copper wires.

**Figure 7:**
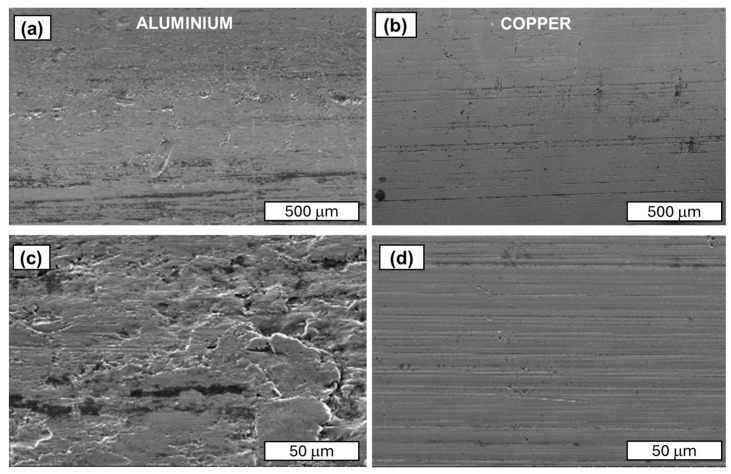
SEM images of the aluminium wire surface: (**a**) magnification ×200 and (**c**) magnification ×2000. SEM images of the copper wire surface: (**b**) magnification ×200 and (**d**) magnification ×2000.

This variation may be related to the chemical purity of the aluminium and copper wires that are used in the tests, as well as the variation in the strength properties of aluminium and copper wires. Aluminium wires have a purity of 99.7% by weight, whereas copper wires have a purity of 99.99% by weight. The typical chemical composition of aluminium that is used for electrical purposes comprises 0.03–0.05% silicon and 0.1 iron by weight, depending on the grade. These elements form various brittle phases of the Fe-Si and Al-Fe-Si type, which act as stress concentration areas in deformed wires. In particular, their presence on the surface of the drawn wire causes a network of microcracks to form during the drawing process when they come into contact with the die’s working cone wall. Consequently, such areas may act as initiation points for the development of fatigue cracks. Additionally, the surface quality of aluminium wires is generally inferior to that of copper wires. A thorough investigation of the wire surfaces, depicted in Figure 7 and the accompanying table, unequivocally demonstrates that the surface quality of Cu-ETP wires surpasses that of EN AW 1370 wires. A multitude of superficial imperfections, including scratches, irregular dents, unevenness, and material discontinuities, have been observed on the surface of aluminium wires. These imperfections are attributed to the presence of a brittle surface layer of aluminium oxide (Al_2_O_3_). Conversely, the surface of copper wires exhibits a reduced propensity for scratches and defects. The surface is covered with a layer of copper oxide, which is brittle and poorly bonded to the copper. Consequently, and in contrast to aluminium oxide, it falls off the base metal during the deformation process. Therefore, the presence of an external layer of copper oxide on the surface of Cu-ETP wires does not pose a problem in terms of surface quality, as is the case with aluminium. There is a higher probability of defects in the form of inclusions and material discontinuities in the analysed aluminium wires, which result in immediate decohesion of the material in the fatigue process.

**Figure 8:**
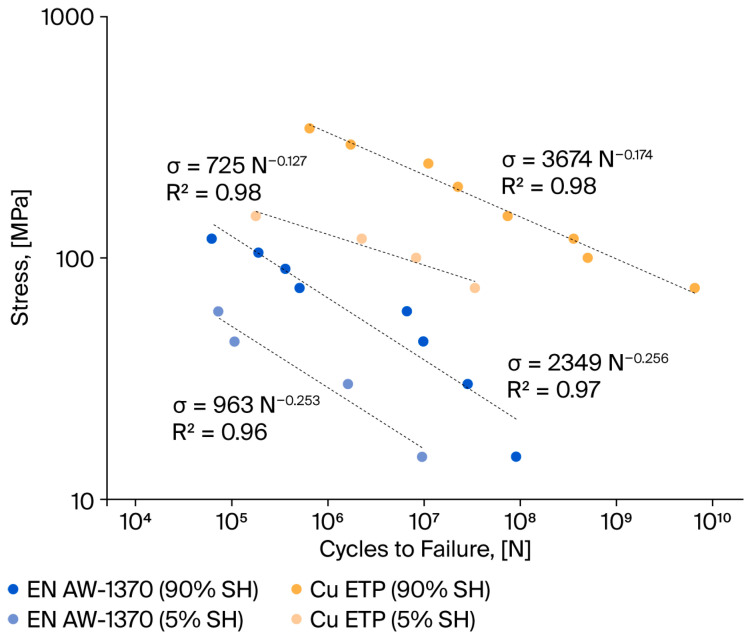
Wöhler diagrams of aluminium and copper wires with different strain hardening levels.

The fatigue test results were averaged, and Wöhler characteristics were determined to quantitatively assess the fatigue strength variation in the wires (see Figure 8). The main result is the higher fatigue strength of Cu wires, which can withstand up to 10^10^ cycles, while the HCF for aluminium wires is in the order of 10^8^ cycles. This is obviously due to the difference in static strength between the two materials. The curves presented herein were developed for stress points within the elastic range, i.e., below the yield strength of the wires. Consequently, for wires in the 5% SH state, the Wohler curve was constructed using a foundation of four stress values. For wires in the 90% SH state, the curve was constructed using a foundation of eight stress values.

The points are generally arranged in parallel, with the results for the copper wires being located over those for the aluminium wires. The fatigue characteristics of aluminium wires in a hardening state approximate the curve representing copper wires in a soft state. Both aluminium and copper wires in a soft state demonstrate low fatigue strength. The maximum number of cycles for aluminium wires was 1.95 × 10^7^, while for copper wires, it was 6.09 × 10^8^.

The data were approximated using a power function (Wöhler equation) of the following form:

(4) $$\sigma_{max}=C\cdot N^{Z}$$

$\sigma_{max}$—max stress during fatigue test;
C—linear coefficient;
N—number of cycles to failure;
Z—power coefficient.

The results of the Wöhler equation parameters are presented in Table 6.

The fundamental difference between these graphs for the two materials is the difference in the slope of the curve, which confirms the value of the power coefficient, Z. For aluminium wires, Z equals −0.253 (5% SH) and −0.256 (90% SH), while for copper wires, it ranges from −0.127 (5% SH) to −0.174 (90% SH). In general, the findings of our fatigue strength analysis show that hard copper wires exhibit better fatigue strength than aluminium wires. However, hard Al wires have a similar fatigue strength to soft Cu wires. At low stress amplitudes, both wires exhibit similar properties, indicating comparable stress ranges for Al and Cu wires. The Z parameter is indicative of the intensity of local stresses in micro-areas of the material (e.g., at grain boundaries and defect concentrations), which determine the rate of damage accumulation at the microscopic level. An elevated Z value is indicative of enhanced dynamics and density with respect to dislocation formation and migration, in addition to accelerated initiation and propagation of microcracks. From a microscopic perspective, the “Z” factor facilitates the establishment of a correlation between the number of cycles to failure that can be observed in the Wöhler diagram and the growth and distribution of lattice defects, as well as the microstructure of the material. Research has demonstrated that an elevated Z factor is indicative of accelerated local microstructure degradation, which in turn corresponds to a reduced fatigue life.

### 3.2. Analysis of Morphology of Fatigue Fractures

**Figure 9:**
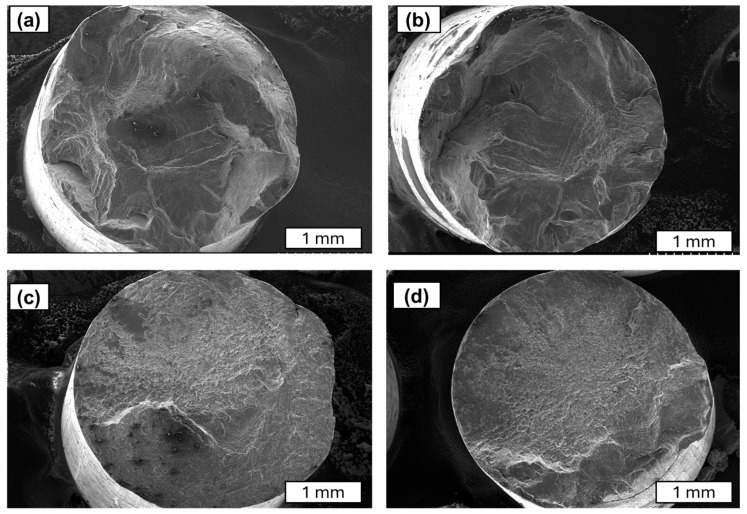
SEM images of the fatigue cracks of aluminium wires after undergoing LCF (**a**,**b**) and HCF (**c**,**d**); magnification 30×.

**Figure 10:**
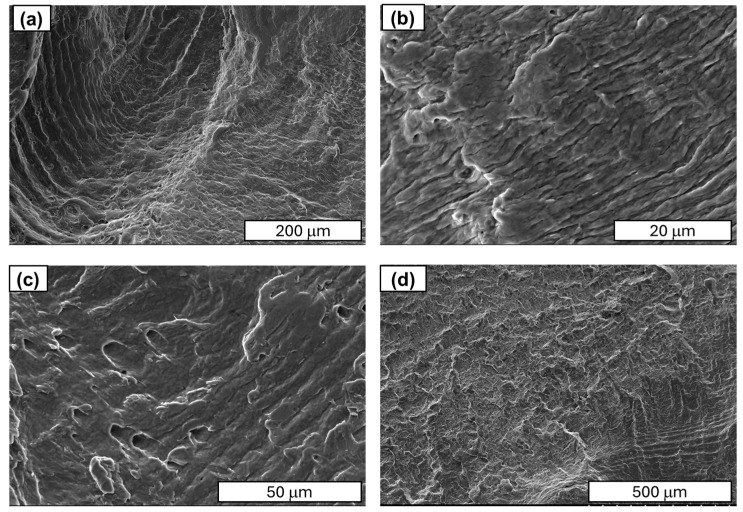
SEM images of the aluminium wire areas with fatigue striations: (**a**) LCF test—magnification ×250; (**b**) LCF test—magnification ×2000; (**c**) HCF test—magnification ×1000; (**d**) HCF test—magnification ×100.

**Figure 11:**
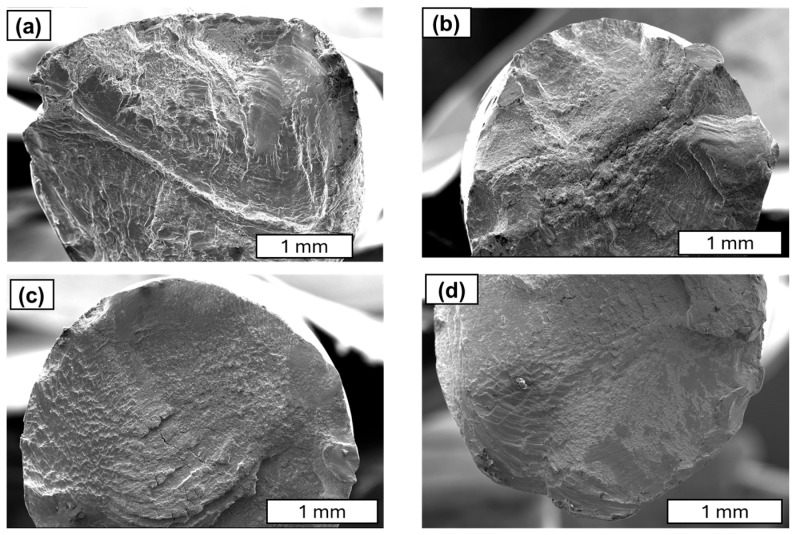
SEM images of the fatigue cracks in copper wires (90% SH) after undergoing LCF (**a**,**b**) and HCF (**c**,**d**); magnification 30×.

**Figure 12:**
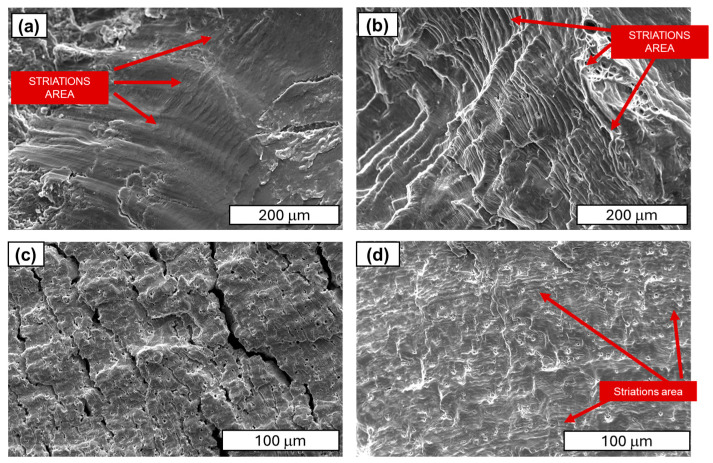
SEM images of the copper wire (90% SH) areas with fatigue striations: (**a**) LCF test—magnification ×250; (**b**) LCF test—magnification ×250; (**c**) HCF test—magnification ×500; (**d**) HCF test—magnification ×500.

Further studies focused on analysing the morphology of fatigue cracks. During fatigue testing, several phases of crack development can be distinguished. The initial phase is characterised by local plastic deformation. The second phase is characterised by the appearance of microcracks. The third phase is characterised by the interlocking of microcracks or microfractures and the formation of a primary fracture. According to the study described in [24], cold-drawn commercially pure aluminium wires show a four-stage fracture pattern, crack initiation, planar crack propagation, 45°–inclined crack propagation, and final rapid fracture, with cracks initially growing along grain boundaries before following maximum shear stress planes. Similar findings have been observed in SEM research. A characteristic feature of fatigue fracture is striations (see Figure 9), which appear as protrusions and indentations that are oriented parallel to the fracture front. The presence of fatigue striations, indicating the movement of the fracture front during each cycle, is a direct indicator of the actual load history (see Figure 9). It has been shown that the presence of fatigue striations, which are traces of crack front movement in each cycle, accurately reflects the actual load history. Striations occur in various forms and are primarily classified as plastic or brittle striations. Figure 9, Figure 10, Figure 11 and Figure 12 present SEM images of damage to the copper and aluminium wires that were tested in this study after being subjected to low-cycle fatigue (LCF) (a and b) and high-cycle fatigue (HCF) (c and d) processes.

A comparison between low-cycle fatigue (LCF) and high-cycle fatigue (HCF) fractures reveals that the surface of the former is significantly more extensive and developed, indicating that the specimen was destroyed during plastic deformation. The fatigue zone exhibits a comparatively minor share, with distinct accumulations in the central part of the fracture, and numerous dents and faults are evident. Conversely, the surface of the high-cycle fracture exhibits a significantly higher proportion of both fatigue damage and the focal zone. The fracture surface exhibits a flattened, smooth, and shiny appearance in certain areas, and characteristic formations for this fracture type, such as fatigue tongues and basins, are discernible. The front lines are distinctly marked on the surface, indicating the direction of the crack, and there are minor faults between the planes (see Figure 10).

Fatigue striations, defined as the traces of the developing crack front’s movement in each cycle, are observed to be parallel to the aforementioned crack front. The striations differ from fatigue lines in terms of size. The presence of a prominent striation may be indicative of a fatigue line or a fault.

A similar morphology of fatigue cracks is observed in copper wires. In the case of LCF, typical plastic cracks with a torn surface are observed, while in the case of HCF, typical fatigue fractures are observed. SEM images at higher magnification demonstrated that the layering observed in the Cu samples was analogous to the layering that was previously documented in aluminium. Furthermore, the analysis revealed the presence of cracks and delamination of the material (see Figure 11 and Figure 12). An analysis of fatigue fractures in aluminium wires revealed that no such cracks occurred.

When analysing striations, it is imperative to consider the widths of the bands, as they are dependent on the material’s mechanical condition and the level of stress experienced during fatigue. Tanaka’s observations in [33] demonstrated that, for a stress amplitude of 100 MPa, the width of the stripes in the heat-treated sample was greater than that in the untreated sample. It is evident that the amplitude of plastic deformation that was exerted on the heat-treated specimen during the fatigue test was greater than that of the untreated specimen under identical stress amplitude conditions. As illustrated in Figure 12, it was determined through analysis of the images that the stations in copper wires with 90% SH are smaller and narrower than those obtained in [33]. Our comparative analysis of copper and aluminium revealed that aluminium’s striations exhibit wider spacing than those of copper (see Figure 10 and Figure 11). The underlying factors that led to this situation are attributable to the increased grain size of the aluminium wires.

**Figure 13:**
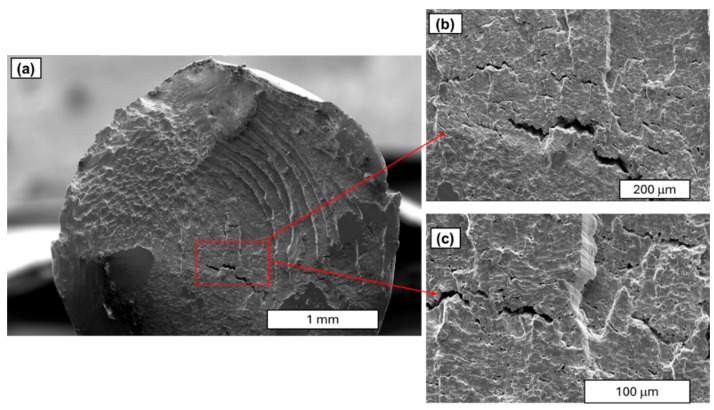
SEM images of copper wires (sample 1, 90% SH) after HCF process: (**a**) magnification ×35; (**b**) magnification ×150; (**c**) magnification ×500.

**Figure 14:**
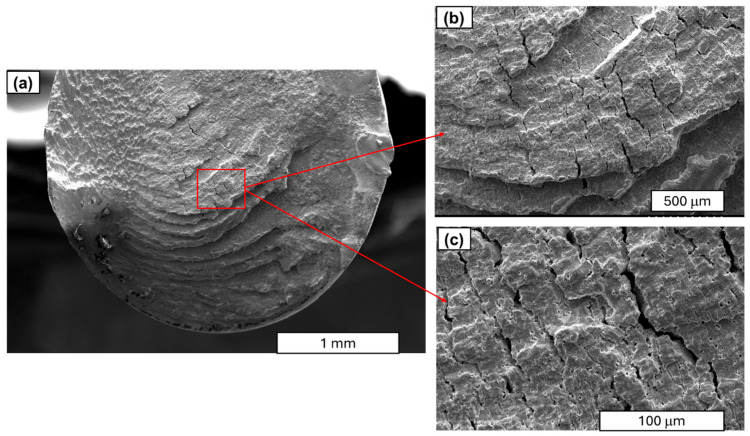
SEM images of copper wires (sample 2, 90% SH) after HCF process: (**a**) magnification ×35; (**b**) magnification ×150; (**c**) magnification ×500.

As in the case of Cu wires, clear cracks are observed in the central part of the Cu wire’s cross-section (see Figure 12c). These fractures are the result of material weakening after fatigue and the final breakage of the sample. This may be the effect of higher levels of bending–torsional stresses in the copper structure. A detailed analysis of the copper wire cracks is presented in Figure 13 and Figure 14.

The figure above illustrates a small, typical fatigue zone with lines, while secondary cracks are clearly visible in the central part of the scrap. The surface is notable for its marked development, as evidenced by the presence of noticeable dents and basin-like formations and tongues, i.e., formations with characteristic shapes. The considered fracture is distinguished by the presence of distinct structural elements, manifesting as fatigue lines and a pronounced perifocal zone surface. In the central part, basin-like formations are visible, accompanied by flat areas located in the cleavage plane of the material. Furthermore, faults between successive planes are also evident.

### 3.3. Analysis of Wire Fatigue Results for Implementation in the Design of Cable Fatigue

The findings of this study raise the question of whether these results can be applied to cable design. A comprehensive understanding of the fatigue strength of a single aluminium or copper wire is instrumental in facilitating the prediction of cable fatigue. However, this understanding is predicated on the consideration of numerous additional data points. Single wires are the foundational element of all conductors and cable systems. The fatigue strength of cables is a multifaceted issue, influenced by numerous factors, including the following: (1) the type of material of the single wire; (2) the type of conductor/cable construction (regular, irregular, monomaterial, or mixed-material wires, e.g., aluminium wires and copper wires or their alloys); (3) the weight and diameter of the conductors; (4) the atmospheric conditions (wind speed, rime, and temperature); and (5) the geometry of overhead lines (span length and height of towers).

In the context of overhead power lines, the cables are subject to various loads during their operation in real working conditions. The loads in question are the result of various factors, including the cables’ weight, the mechanics of their span, and the effects of wind action. Consequently, the wires within the cable structure are subjected to stretching, twisting, and bending. It is imperative to acknowledge that the cable’s multilayer configuration facilitates interwire interactions during periods of fatigue. The mechanism of the fatigue power of cables involves the repetitive movement of the cable, which engenders relative movements on the contact surfaces of the following components: (a) wire-to-wire, (b) wire-to-terminal, and (c) wire-to-clamp. The result of repetitive displacements on the contact surfaces of wires in distinct layers of the cable is a phenomenon of wire surface degradation known as “fretting corrosion” or “friction corrosion” [6,8,9].

The catalysts of friction corrosion can be categorised into three distinct categories: (a) an increase in the load on the wire surface; (b) an increase in temperature; and (c) an increase in the intensity of the relative movement on the wire contact surface. Friction corrosion has been identified as the primary cause of accelerated fatigue cracking. As previously mentioned, the factors described herein decrease the fatigue endurance of cables. This indicates that conductors experience fatigue failure at a significantly lower number of cycles than the wires from which they are composed. It is reasonable to continue studying the fatigue of aluminium and copper wires in various mechanical states (in various states of hardening) based on the experimental research findings. Results obtained in this manner will facilitate an assessment of the impact of the hardening state of copper and aluminium on the change in their fatigue strength and will clearly indicate the role of grain size and texture, as in [34,36].

It is imperative that research is conducted to ascertain the process conditions and identify crack initiation in aluminium and copper wires. This should be undertaken in conjunction with the elucidation of the crack growth mechanism. Such studies are commonly utilised in the context of steel wires, as evidenced in [13]. Furthermore, the findings obtained from this study provide a foundation for the execution of a comprehensive research programme, the objective of which is to investigate the relationship between the fatigue strength of a single wire and the fatigue strength of a cable. At present, numerical simulations are the most prevalent, with a significantly lower number of experimental studies, such as those documented in [12,13,24,25].

## 4. Conclusions

In this study, we present a comparative analysis of the fatigue strength of copper and aluminium conductors utilised in overhead lines.

1. The fatigue strength analysis encompassed the preparation of classic S-N diagrams in coordinate systems based on the bending stress amplitude and number of cycles. The Wöhler characteristics for Cu and Al intersect and differ in terms of the slope of the logσ-logN system. The curve representing aluminium wires lies slightly below the curve representing Cu-ETP wires. The fundamental distinction between these two categories is predicated on the disparity in their respective slopes, which is evidenced by the power coefficient Z, as delineated by the Wöhler equation. For aluminium wires, the Z factor is −0.253 (5% SH) and −0.256 (90% SH), while for copper wires, Z is −0.127 (5% SH) and −0.174 (90% SH). The findings of these experiments demonstrated that the absolute value of Z for aluminium wires is nearly double that of copper wires. Research has demonstrated that an elevated Z factor is indicative of accelerated degradation of the local microstructure, which, in turn, corresponds to reduced fatigue life.
2. The most significant conclusion pertains to the enhanced fatigue strength exhibited by Cu-ETP wires, a phenomenon that can be attributed to their distinctive microstructural characteristics. This conclusion is applicable to copper in its mechanical state: 90% SH and 5% SH. From the perspective of microscopic fatigue fracture mechanisms, which contribute to the enhanced fatigue life of copper wires, the most salient factors are as follows: (a) the microstructure, which is characterised by homogeneity and the presence of fine grains; (b) the higher chemical purity of copper; and (c) the capacity of copper to effectively absorb and dissipate dislocations, which is the result of its higher plasticity.
3. The primary factors contributing to the reduction in the fatigue strength of aluminium include lower static strength, larger grains in the aluminium structure, and poorer wire surface quality. Aluminium wires are characterised by their substantial columnar grains, with an average size of 100 micrometres. A thorough examination of the surface of aluminium and copper wires using scanning electron microscopy (SEM) reveals a substantial prevalence of surface defects in aluminium wires. These surface defects may play a pivotal role in accelerating the initiation of crack formation.
4. The results of the SEM test demonstrate a clear distinction in the fatigue damage susceptibility of aluminium and copper wires. However, it should be noted that the mechanism of fatigue crack formation varies depending on the stress level at which the test is performed. In the case of cracks that are formed in the LCF range, a developed crack surface with clear plastic deformation is usually observed. In contrast, in the case of HCF, fatigue cracks in both materials manifest as smooth surfaces and exhibit characteristic striations. It is worth noting that in both cases, the observed defects are consistent with the formation of fatigue cracks. In the case of copper wires, the fatigue damage zone is characterised by a smoothed, crusty surface. A distinctive attribute of fatigue cracks in hardened copper wires is the manifestation of visible cracks along the grain boundaries, which function as indicators of the fatigue crack surface. In the case of aluminium wires, the fatigue damage zone exhibited a reduced surface area, while the temporary damage zone demonstrated a surface characteristic of elastic cracks. In contrast to the visible individual cracks that were found on the surface of the fatigue crack in copper wires, no such visible cracks were found on the surface of the fatigue crack under investigation.
5. In the case of LCF, for both aluminium and copper, the fracture is characterised by an open surface, with clear signs of plastic deformation. Conversely, the HCF analysis of the fractures revealed discrepancies in the fatigue damage mechanism of aluminium and copper wires. Furthermore, the presence of material discontinuities, manifesting as isolated cracks, was detected in copper wires within the designated fatigue damage area. These cracks manifest in copper wires subsequently to HCF processes. No such cracks were observed in the aluminium wires.

## Figures and Tables

**Table 1:** Chemical composition of the aluminium (EN AW-1370) used in the test.

Material	Chemical Composition [% by Weight].								
	Al Min.	Si	Fe	Cu	Mn	Mg	Cr	Zn	Ga
Aluminium	97.72	0.03	0.10	0.04	0.002	0.001	0.001	0.03	0.01

**Table 2:** Chemical composition of the copper (Cu-ETP) wire used in the test.

Material		Chemical Composition [% by Weight].												
	Cu	O_2_	Ag	Bi	Pb	Sb	As	Fe	Ni	Sn	Zn	S	Se	Te
Cu-ETP	99.99	0.019	0.0009	0.00001	0.00007	0.00006	0.00006	0.000017	0.000017	0.000003	0.00008	0.000025	0.000001	0.000002

**Table 3:** Mechanical properties of the aluminium and copper wires used in the tests.

Material	Diameter d_0_	Diameter d_1_ [mm]	Engineering Strain	UTS [MPa]	YP [MPa]	Elongation [%]
	[mm]		[%]			
EN AW-1370	9.50	3.00	90	168	139	1.54
EN AW-1370	3.08	3.00	5	66	52	26.0
Cu-ETP	9.50	3.00	90	433	413	3.50
Cu-ETP	3.08	3.00	5	245	170	28.4

**Table 4:** Applied stress values in fatigue test used for obtaining Wöhler curves.

Material	Applied Max Bending Stress [MPa]							
EN AW-1370	120	105	90	75	60	45	30	15
Cu-ETP	344	295	246	197	149	120	100	75

**Table 5:** Results for the copper and aluminium wires (5% and 90% strain hardening) from the rotary bending fatigue test.

Test No	Cu Wires			Al Wires		
	Bending/Rotary Stress, MPa	Number of Cycles to Failure		Bending/Rotary Stress, MPa	Number of Cycles to Failure	
		5% (SH)	90% (SH)		5% (SH)	90% (SH)
1	344	1.50 × 10^3^	3.10 × 10^5^	120	1.80 × 10^3^	2.58 × 10^4^
	295	1.00 × 10^3^	2.88 × 10^6^	105	1.00 × 10^4^	1.44 × 10^5^
	246	5.28 × 10^4^	6.66 × 10^6^	90	7.20 × 10^3^	8.88 × 10^4^
	197	5.85 × 10^4^	2.25 × 10^7^	75	4.00 × 10^4^	2.70 × 10^5^
	149	1.60 × 10^6^	7.40 × 10^7^	60	2.30 × 10^4^	5.67 × 10^6^
	120	2.20 × 10^6^	3.60 × 10^8^	45	6.93 × 10^5^	9.80 × 10^6^
	100	5.71 × 10^6^	2.24 × 10^8^	30	1.88 × 10^6^	2.58 × 10^7^
	75	3.09 × 10^7^	6.00 × 10^9^	15	1.05 × 10^7^	9.00 × 10^7^
2	344	2.20 × 10^3^	5.93 × 10^5^	120	1.40 × 10^3^	3.87 × 10^4^
	295	2.52 × 10^3^	5.76 × 10^6^	105	2.04 × 10^5^	1.87 × 10^5^
	246	8.40 × 10^4^	8.88 × 10^6^	90	1.92 × 10^4^	2.66 × 10^5^
	197	8.55 × 10^4^	2.25 × 10^7^	75	3.43 × 10^4^	3.87 × 10^5^
	149	1.49 × 10^6^	7.40 × 10^7^	60	9.80 × 10^4^	5.92 × 10^6^
	120	2.21 × 10^6^	3.60 × 10^8^	45	5.05 × 10^5^	9.80 × 10^6^
	100	6.72 × 10^6^	3.36 × 10^8^	30	1.48 × 10^6^	2.75 × 10^7^
	75	3.14 × 10^7^	6.22 × 10^9^	15	1.05 × 10^7^	9.70 × 10^7^
3	344	2.20 × 10^3^	8.51 × 10^5^	120	1.78 × 10^3^	5.94 × 10^4^
	295	5.04 × 10^3^	1.15 × 10^7^	105	7.00 × 10^4^	1.99 × 10^5^
	246	9.12 × 10^4^	1.55 × 10^7^	90	8.60 × 10^3^	3.82 × 10^5^
	197	1.80 × 10^5^	2.25 × 10^7^	75	3.00 × 10^3^	5.94 × 10^5^
	149	1.92 × 10^6^	7.40 × 10^7^	60	2.20 × 10^4^	6.44 × 10^6^
	120	2.22 × 10^6^	3.60 × 10^8^	45	3.80 × 10^5^	9.86 × 10^6^
	100	7.56 × 10^6^	6.73 × 10^8^	30	1.60 × 10^6^	2.93 × 10^7^
	75	3.14 × 10^7^	7.00 × 10^9^	15	1.00 × 10^7^	8.02 × 10^7^
4	344	4.40 × 10^3^	8.26 × 10^5^	120	2.00 × 10^3^	7.74 × 10^4^
	295	3.84 × 10^3^	1.44 × 10^7^	105	1.28 × 10^5^	2.30 × 10^5^
	246	6.00 × 10^4^	1.33 × 10^7^	90	2.30 × 10^4^	7.10 × 10^5^
	197	1.26 × 10^5^	2.25 × 10^7^	75	9.77 × 10^4^	7.83 × 10^5^
	149	2.13 × 10^6^	7.40 × 10^7^	60	1.48 × 10^5^	8.63 × 10^6^
	120	2.30 × 10^6^	3.60 × 10^8^	45	7.00 × 10^5^	9.99 × 10^6^
	100	1.31 × 10^7^	7.85 × 10^8^	30	1.51 × 10^6^	3.13 × 10^7^
	75	4.18 × 10^7^	6.90 × 10^9^	15	1.10 × 10^7^	9.62 × 10^7^

**Table 6:** The parameters of the Wöhler curve of the Al and Cu wires.

Lp.	Material	Strain Hardening [%]	C [−]	Z [−]
1	EN AW 1370	5	963	−0.253
2	EN AW 1370	90	2349	−0.256
3	Cu-ETP	5	725	−0.127
4	Cu-ETP	90	3674	−0.174

## Data Availability

The original contributions presented in this study are included in the article. Further inquiries can be directed to the corresponding author.
